# Government Health Expenditure and Public Health Outcomes: A Comparative Study among EU Developing Countries

**DOI:** 10.3390/ijerph182010725

**Published:** 2021-10-13

**Authors:** Mihaela Onofrei, Anca-Florentina Vatamanu, Georgeta Vintilă, Elena Cigu

**Affiliations:** 1 Faculty of Economics and Business Administration, Alexandru Ioan Cuza University, 700505 Iasi, Romania; onofrei@uaic.ro (M.O.); elena.chelaru@uaic.ro (E.C.); 2 Department of Finance, The Bucharest University of Economic Studies, 010374 Bucharest, Romania; vintilageorgeta@yahoo.fr

**Keywords:** health outcomes indicators, demographic vulnerabilities, socio-economic vulnerabilities, life expectancy, infant mortality

## Abstract

The aim of this paper was to empirically analyze the relationship between public health expenditure and health outcomes among EU developing countries. Using regression analysis and factor analysis, we documented that public health expenditure and health outcomes are in a long-run equilibrium relationship and the status of health expenditure can improve life expectancy and reduce infant mortality. Secondarily, we studied how the status of good governance, health care system performance, and socioeconomic vulnerabilities affect the public health’s outcomes in the selected countries. We found that the effectiveness of health and the way to reduce infant mortality or to improve life quality is directed conditioned by good governance status. Moreover, the consolidation of health care system performance directly improves the quality of life among EU developing countries, which indicates that public policymakers should intervene and provide political and financial support through policy mixes.

## 1. Introduction

The run-up of the global financial crisis deepened economic shocks, poses a threat to health system performance, and caused distortions in the allocation of public resources. The people’s needs for health increased and public health outcomes indicators are influenced by the dimension of public reforms and the related governance framework. Typically, in crisis periods, governments’ expenditures tend to increase and citizen’s expectations are overly optimistic. When the process is reversed by both endogenous and exogenous factors, public health reforms are designed and planned considering the diversity of the healthcare system that is directly reflected in the degree of public health expenditure. As a consequence, the impact on health outcomes can vary between countries. As the quality of public health expenditure is reflected in health outcomes among countries, the co-movement and causal linkages between public health outcomes and healthcare expenditure depend on governments implication in providing a quality life for its citizens through a good health system, meaning that in times of vulnerabilities, the pressure is higher and require solid strategies in terms of revenue and expenditure [1]. The unprecedented financial stress related to the battle against the COVID-19 pandemic has led to a sharpening of spending conditions and most of the studies reveal that healthcare capacity faces major challenges and vulnerabilities [2,3,4]. However, Noura [5] noted that the gaps in health care systems already existed prior to the COVID-19 pandemic and the inefficiency in resource allocation was just easier to explain in such a time of vulnerabilities. In other words, ex-ante financial disturbances are a key determinant of health care system risk and an understanding of cyclical movements of financial indicators is a sine qua non for a correct design of public health care policies.

In their paper, Makina and Laytonb [6] revealed that the governments around the world responded to the COVID-19 crisis by aggressively deploying fiscal policy to boost health expenditure and the related public debt levels will put higher pressure on the governments around the world and will require concrete measures for fiscal consolidation. The capacity to generate new public reforms capable to improve public health performance and positively impact the overall well-being depends, of course, on the level of economic growth, because it is quite difficult to increase the money spent on health finance if there is not enough fiscal space to maneuver [7,8].

In line with the last point of view, the economic literature recognizes the benefits of good health and establishes a direct relationship between the status of health and the development of countries [9,10,11,12]. It is imperative for all countries to appropriately invest in their health sector to realize the linkage between longevity and benefits of the economy, but as Mohammad et al., 2018 revealed in their paper, the amount of spending should be managed by proper governance and adequate policies capable of streamlining public sector health funds.

It is recognized that research reveals mixed results related to government spending on health but leans toward positive outcomes from increased public spending [13,14,15]. There are two common approaches used to determine the implication of government spending on public health outcomes. The first one relies on Grossman’s product (which reveals the aggregate health production function) and considers health as a capital good that can be affected over time and that depends on several endogenous and exogenous variables [16,17]. The second rationale was developed by Zweifel and Breyer [18] and considers health as an output of the entire health care system, which is influenced by the related inputs and investigates, for instance, the relationship between health care expenditure (considered as inputs) and health outcomes (considered as outputs).

While it is widely acknowledged that the theoretical insights document a range of effects, from no impacts, to limited, and to the significant impact of public health expenditure on health outcomes, due to lack of studies performed on the profile of EU countries, we empirically analyzed the relationship between public health expenditure and health outcomes among EU developing countries. The study provides new evidence on a panel of EU developing countries and based on regression analysis and factor analysis, empirically analyzed the relationship between public health expenditure and health outcomes. The study has broader coverage and represents an important contribution to the literature by the fact that, in order to explain the variations in death rates across countries, it included three categories of factors: health, demographic, and socio-economic vulnerabilities indicators. Additionally, the effects of health expenditure on these categories of three factors were investigated, and based on the methodological approach, the endogeneity issues were addressed. We documented that public health expenditure and health outcomes are in a long-run equilibrium relationship and the status of health expenditure can improve life expectancy and reduce infant mortality. The remainder of the paper is structured as follows: In Section 2 we detail the methodology we employ, in Section 3 we present the empirical findings and discussion, and in Section 4 we conclude the study.

## 2. Empirical Framework and Methodology

The retrospective of theoretical insights documents a range of effects, from no impacts, to limited, and to the significant impact of public health expenditure on health outcomes. For example, some authors bring into focus the significant relationship between health expenditures and health indicators outcomes among countries with different health care systems [19,20,21], and others explore the effects of health expenditure on health outcomes in Sub-Saharan Africa and reveal the implication of health expenditure on reducing mortality rates and improving life expectancy at birth [22]. On the other hand, Kulkarni [23] validated the profile of the BRICS countries and found that alone, simply increasing health expenditure cannot positively impact health outcomes and a better quality of the financial system and related mechanisms is necessary, this being also supported by Kim et al. [24] in their paper entitled “Income, financial barriers to health care and public health expenditure: a multilevel analysis of 28 countries”, which also highlighted that health system financing should be better planned and capable of managing inequalities in access to health. Contrary to the above-mentioned literature insights, Yaqub et al. [25] brought into discussion the implication of corruption status, contending that public health expenditure has a negative effect on infant mortality and revealed, on the profile of Nigeria, that the success in reducing mortality rates and lowering the infant mortality depends on the implication in reduced considerably the level of corruption. Regarding the profile of EU countries, there is a lack of studies that explore the relationship between government health spending and public health outcomes, though we found some literature that reveals the relationship between health spending and foreign direct investment [26], and others that analyze the state of health spending in times of crisis [27,28]. Therefore, the main rationale for conducting current research on the profile of EU developing countries was based on the existing gap in the literature.

Following the literature insights mentioned in previous section, the ordinary least squares (OLS) and the two-stage least squares (2SLS) estimators were employed for analyzing the relationship between government health expenditure and public health outcomes, and the studies were both cross-sectional and panel data types. However, by retrospective analysis and above-mentioned literature insights, we realize that the approaches are blurred, and a lot of the variables employed in the two approaches are similar. Therefore, to avoid the methodological problems and to develop the study in line with literature validation, we employed panel data analysis and factor analysis, and we empirically analyzed the relationship between public health expenditure and health outcomes among EU developing countries over the period of 2000–2019. As a first step, the objective to analyze the implication of government health expenditure on public health outcomes requires the establishment of public health outcomes indicators. We followed the literature insights [29,30,31,32,33] and we used two public health outcomes indicators, namely life expectancy at birth and infant mortality. Secondarily, in order to explain the variations in death rates across countries, we used three categories of factors: health, demographic, and socio-economic vulnerabilities indicators (see Appendix A for a detailed description of each variable according to the related code and original source).

The first factor (*F*1) is related to the quality of life and dimension of public governance and includes eleven sub-indicators: Real GDP growth rate (RGGR), age dependency ratio (ADR), domestic general government health expenditure (DGGE), human capital index (HCI), nurses and midwives (NM), government effectiveness (GE), control of corruption (CCOR), political stability and absence of violence/terrorism (PS), regulatory quality (RQ), rule of law (RL), and people using safely managed sanitation services (PUSS). Concerning the variables used, we found validation in light of the approaches commonly applied in previous research, it being widely recognized that the improvements in socioeconomic performance, healthcare, employment, and politics are positively correlated with the longevity and direct impact the status of infant mortality [34,35]. Moreover, the results provided by Helliwell et al. [36] suggested that changes in governance quality within a policy-relevant time horizon can lead to significant changes in the quality of life and other previous research even confirm that people are more satisfied with their lives in countries with high-quality level of governance [37,38,39]. In an extensive documentation of the studies on the impact of the enhancement of quality of life and good administration on infant mortality, it is revealed that both well-being and good governance frameworks influence the trend of infant mortality [40,41,42,43].

Following the previous literature insights on the international comparison of health performance, we found that health outcomes and wellbeing are critical drivers of sustainable development, and the status of health care services depends on the system performance [44,45,46,47]. Thus, we included in our analysis the second factor (*F*2) named health care system performance, which incorporates five sub-indicators: the number of practicing physicians per 1000 Population (PH), Available hospital beds for the care of admitted patients (ABH), Age dependency ratio, young (AD-% of working-age population), Premature deaths, % total premature deaths ambient particulate matter (PDAP), and death rate, crude (DR per 1000 people). According to Kroneman and Siegers [48] at the EU level, the reduction of care hospitals represents a measure implemented to limit expenditure and David et al. [49] even confirmed that hospital bed reduction and multiple-system reform affect patient mortality. Leiyu Shi [50] examined the relationship between the availability of primary care and longevity, suggesting a significant implication of the number of specialty physicians and total mortality.

According to the literature insights, when we talk about growth in human capital, we refer to two important dimensions, education, and health, meaning that those variables positively affect per-capita income in the long run [51,52,53]. The healthcare system is an important determinant of sustainable development and should always be the core of the development of a nation, which is why some authors place a special emphasis on the effects of health expenditure on life expectancy and conclude that the expenditure growth can increase longevity. Jakovljevic et al. [13] and others such as Rahman et al. [54], using the World Bank data set for 15 countries over 20 years (1995–2014), revealed that health expenditure, including public and private, significantly reduced infant mortality rates. Therefore, the last factor included in the analysis (*F*3) captured socio-economic vulnerabilities, and included four sub-indicators: Standardized Gini variable, which measures the income inequality (*GINI*), unemployment, total (*UL* % of the total labor force), population ages 65 and above (% of total population-POP), and labor force with intermediate education (*LFIE*). The human life span is relatively fixed, but improving the quality of life through health care quality could reduce the need for medical care. Nevertheless, House et al., 1990 argued that there is a direct relationship between age, socioeconomic status, and health outcomes, this point of view being also validated by other contemporary research conducted in the context of COVID-19 pandemic risk, which reveals the implication of the societal risk factors and economic vulnerability on mortality rate [55,56,57].

To assess the implication of government health expenditure on public health outcomes, we used ordinary least-squares regression model (OLS) analysis and factor analysis methods. The model includes relevant explanatory variables that influence the level of health outcomes, several categories of public expenses as proxies for the government actions towards health protection and in order to eliminate the problems of skewed distribution, to exclude the orthogonal relationship between components and generate independent components, Factors 1, 2, and 3 were computed based on exploratory factor analysis methodology and we tested the implication of three categories of factors: health, demographic, and socio-economic vulnerabilities indicators. We used as dependent variable two public health outcomes indicators, namely life expectancy at birth and infant mortality and, in order to explain the variations in death rates across countries, we used three categories of factors: health, demographic, and socio-economic vulnerabilities indicators. We used the OLS and factor analysis approach with the following specification:

(1) $$\begin{array}{ll} LE{\;}_{it}= & c_{0}+c_{1}\times CHE_{i,t}\\ & +c_{2}\times \;GDPCAP_{i,t}+c_{3}\times GINI_{i,t}+c_{4}\times UL_{i,t}+c_{5}\times F1_{i,t}\\ & +c_{6}\times F2_{i,t}+c_{7}\times F3_{i,t}+u_{i,t},\; \end{array}$$

where *i* and *t* indicate the country and year for each variable. The dependent variable $LE{\;}_{it}$ represents a key metric for assessing population health and indicates life expectancy at birth, total (years). The independent variables are displayed in Appendix A, and include public health expenditure % of total health expenditure (*CHE*), real GDP per capita (*GDPCAP*), Gini coefficient (*GINI*), an indicator which measures the degree of inequality in countries’ health and the deviation of income distribution from totally equal distribution, unemployment, total % of total labor force (*UL*), the quality of life and dimension of governance (*F*1), Health care system performance (*F*2), and Socioeconomic vulnerabilities (*F*3). The last three factors, *F*1, *F*2, and *F*3 were computed based on factor analysis.

(2) $$\begin{array}{c} MRI{\;}_{it}=c_{0}+c_{1}\times CHE_{i,t}+c_{2}\times \;GDPCAP_{i,t}+c_{3}\times GINI_{i,t}+c_{4}\times UL_{i,t}+\\ c_{5}\times F1_{i,t}+c_{6}\times F2_{i,t}+c_{7}\times F3_{i,t}+u_{i,t}, \end{array}$$

where *i* and *t* indicate the country and year for each variable. The dependent variable $MRI{\;}_{it}$ represents a key metric for infant mortality and indicates the number of children dying before reaching one year of age, expressed as a rate per 1000 live births in a given year. The independent variables are analogous to those indicated in Equation (1). The list of the examined countries includes Bulgaria, Croatia, Czech Republic, Estonia, Hungary, Latvia, Lithuania, Poland, Romania, Slovakia, and Slovenia. Factors 1, 2, and 3 were computed based on exploratory factor analysis methodology, a method that avoids the problems of skewed distribution and excludes the orthogonal relationship between components and generate independent components. Factor analysis finds a few common factors (say, *q* of them) that linearly reconstruct the p original variables:

(3) $$y_{ij}=Z_{i1}b_{1j}+Z_{i2}b_{2j}+Z_{i3}b_{3j}+\ldots Z_{iq}b_{qj}+e_{ij},\;$$

In which case, $y_{ij}$ represents the value of the *i*th observation of the *j*th variable, $Z_{ik}$ represents the *i*th observation on the kth common factor, $b_{qj}\;$ represents the set of linear coefficients named the factor loading, and finally, $e_{ij}\;$ represents the *j*th variables unique factor. The independent variables are displayed in Appendix A. The fixed-effects model has the following form:

(4) $$Y_{i,t}=\alpha_{i}+X_{i,t}\times \beta +\varepsilon_{i,t,}$$

$Y_{i,t}$ represents the dependent variable for country *i* at time *t*, $\alpha_{i}$ represents an unknown country-specific constant, $X_{i,t}$ indicates the time-variant regressor matrix, and $\varepsilon_{i,t,}$ is the error term; in order to validate the appropriateness of the fixed-effects model, the Hausman test was performed

## 3. Empirical Findings and Discussion

Table 1 summarizes the results of estimating Equations (1) and (2) for the influence of public health expenditure on health outcomes among EU developing countries. The methodological approach includes two separate models with two dependent variables named life expectancy (see model 1) and infant mortality (see model 2). We checked the appropriateness of fixed-effects estimation by running the Hausman test, and the results provided in Table 2 reveal that the fixed effect model is to be used. The variables are validated by previous research [29,30,31,32,33] and represent the most common public health outcomes indicators. The results of the mixed-effect model show a positive relationship between government health expenditure and longevity, measured by life expectancy at birth. According to the fixed-effects model, an increase in health expenditures is associated with increase in life expectancy, and this effect is statistically significant at the 0.5% level. In other words, an increase in the overall public health spending reduces the number of the overall mortality level of a population. The results suggest that a one percent increase in public health expenditure decreases the infant mortality rate by 0.64 %. Thus, the results satisfy the viewpoint of Rahman et al. [54], who sees the dimension of public health expenditure as an opportunity to improve the health status of the population.

Regarding the implication of economic output of countries, we found that the economic performance positively affects the well-being of EU developing countries, and a higher real GDP growth rate is related to higher life expectancy. Additionally, when we test the implication of income on infant mortality rate, the results showed a negative relationship, meaning that the higher the economic performance of countries, the lower the number of children dying before reaching one year of age (expressed as a rate per 1000 live births in a given year). Our results confirm, on the one hand, the study performed by Zaman et al. [58] who revealed that at the individual level, income had a direct influence on health spending, and on the other hand, the viewpoint of Blazquez-Fernández et al. [59] who considered that per-capita income can improve health outcomes. The results of the fixed-effects panel model suggest a strong positive relationship between the economic growth and the level of life expectancy.

The results obtained for the other variables presented in Table 1. reveal an expected sign for the status of country inequality measured by the *GINI* index and suggest a negative relationship between country inequality and life expectancy. It seems that the higher the deviation of income distribution from a totally equal distribution, the lower the life expectancy rate. Similar results are provided by authors [60,61], who indicated that researchers must focus on examining inequality in life expectancy for judging the performance of communities regarding the length of life. The unemployment rate (*UL*) is significant and positively correlated with life expectancy and has a negative impact on infant mortality.

The results for life expectancy are in line with those provided by Granados and Diez Roux [62], who pointed out that the improvements in health are positively correlated with increases in the unemployment rate and contrary to those provided by Singh and Siahpush [63], who tested the relationship between unemployment and life expectancy in the United States and found that life expectancy was lower in areas with higher unemployment rates. Regarding the status of results for infant mortality (MRI), the literature suggests that an increase in the local unemployment rate is correlated with a statistically significant increase in the possibility of having a low birthweight baby, weighing less than 2500 g (see the study performed by Kaplan et al. [64] on the profile of American Community).

Further, following the literature, insights which suggest that the process of analyzing the evolution of public expenditure involves the necessity to study the influencing factors, economic, social, political, and military [65], we tested the implication of three categories of factors: health, demographic, and socio-economic vulnerabilities. Factor 1, 2, and 3 were computed based on exploratory factor analysis methodology and, as can be seen in Table 2 and Table 3, each variable was given a ‘uniqueness’ score and the first three factors (factor 1, factor 2, and factor 3) explained 85% of the total variance.

The results showed that the quality of life and dimension of governance (*F*1) was a significant statistic and has a negative impact on infant mortality. Undoubtedly, one possible explanation for this result is that many governments through improving the dimension of governance directly affect health outcomes and consolidate the status of wellbeing, thus decreasing infant mortality. These results agree with previous findings, showing that quality of government matters in public health and it is a gap in the literature, argued by the fact that most of the papers deal with economic, social, and political factors, but avoid the study of governmental factors [66]. In terms of exposure, we also found that the effectiveness of health and the way to reduce infant mortality or to improve life quality is conditioned by good governance status and the consolidation of health care system performance directly improves the quality of life among EU developing countries. Overall, the results enlarge our knowledge of the implications of government health expenditure on public health outcomes and indicate that in order to consolidate the status of public health, public policymakers should intervene and provide political and financial support through policy mixes.

## 4. Concluding Remarks

The availability of public financial resources represents an important condition for the performance of the health system. The run-up of the global financial crisis deepening the economic shocks increases people’s needs for health, poses a threat to health system performance, and caused distortions in the allocation of public resources. Using regression analysis and factor analysis, we investigated the relationship between public health expenditure and health outcomes among EU developing countries. The paper contributes to related literature through the expansion of the research concerning the evolution of health outcomes and the status of public health expenditure in EU developing countries. The study has a broader coverage and represents an important contribution to the literature by explaining the variations in death rates across countries and including three categories of factors: health, demographic, and socio-economic vulnerabilities indicators. Additionally, the effects of health expenditure on these categories of three factors were investigated and based on the methodological approach, the endogeneity issues were addressed. We studied how the status of good governance, health care system performance, and socioeconomic vulnerabilities affect the public health’s outcomes in the selected countries, and we found that public health outcomes indicators are influenced by the dimension of public reforms and related governance framework. In this particular sample, we also found a strong positive relationship between government health expenditure and longevity, measured by life expectancy at birth. An increase in the overall public health spending reduces the overall mortality level of a population. The results suggest that one percent increase in public health expenditure is associated with a decrease in infant mortality rate by 0.64%. Economic performance positively affects the well-being of EU developing countries, and a higher real GDP growth rate is related to higher life expectancy. Additionally, our results confirmed that income had a direct influence on health spending or could improve health outcomes and, as expected, country inequality measured by the *GINI* index was negatively correlated with life expectancy. Our study suggests the need for health policymakers in EU developing countries to implement active strategies that reduce the death rate and consolidate the wellbeing of communities, to intervene and provide political and financial support through policy mixes.

## Figures and Tables

**Table 1:** The results of the mixed-effect model.

Variables	Model 1 (LE)			Model 2 (MRI)		
	Pooled OLS	Random Effect	Fixed Effect	Pooled OLS	Random Effect	Fixed Effect
CHE	0.357 (2.85) **	0.596 (4.11) **	0.605 (4.13) **	−0.194 (1.49)	−0.129 (0.75)	−0.115 *** (0.64)
GDPCAP	0.000 (8.12) **	0.000 (7.54) **	0.000 (7.52) **	−0.000 (3.06) **	−0.000 (3.27) **	−0.000 (3.26) **
GINI	−0.062 (1.71)	−0.174 (3.79) **	−0.182 (3.94) **	−0.057 (1.51)	0.029 (0.54)	0.037 (0.65)
UL	0.018 (0.68)	0.091 (4.07) **	0.102 (4.63) **	−0.084 (3.14) **	−0.048 (1.75)	−0.056 (2.05) *
F1	−0.032 (0.15)	−0.250 (0.82)	−0.652 (1.93)	−2.522 (11.13) **	−1.779 (5.10) **	−0.965 (2.32) *
F2	−0.236 (1.92)	0.075 (0.59)	0.047 (0.36)	0.169 (1.32)	0.088 (0.59)	0.210 (1.31)
F3	−0.385 (3.15) ***	1.375 (4.99)	−2.098 (6.39) **	0.024 (0.19)	−1.062 (3.55) **	−2.198 (5.43) **
Cons	71.281 (42.53) **	73.201 (45.08) **	73.358 (47.65) **	11.232 (6.44) **	7.836 (4.05) **	7.567 (3.99) **
Hausman		31.48 ***			18.84 ***	
*N*	213	213	213	213	213	213
R2	0.61	0.72	0.73	0.43	0.51	0.68

Source: research results. Notes: the results include the coefficient of variable and *t* statistic results in parentheses; *** *p* < 0.01, ** *p* < 0.05, * *p* < 0.1.

**Table 2:** The results of factorial analysis of the main components for estimating the three main factors.

Factor	Eigenvalue	Difference	Proportion	Cumulative
Factor1	5.839	2.300	0.349	0.349
Factor2	3.539	0.647	0.211	0.560
Factor3	2.892	1.546	0.173	0.733
Factor4	1.346	0.197	0.080	0.813
Factor5	1.148	0.137	0.069	0.882
Factor6	1.011	0.361	0.060	0.942
Factor7	0.650	0.285	0.039	0.981
Factor8	0.366	0.148	0.022	1.003
Factor9	0.218	0.082	0.013	1.016
Factor10	0.136	0.099	0.008	1.024
Factor11	0.037	0.022	0.002	1.026
Factor12	0.015	0.015	0.001	1.027
Factor13	0.000	0.012	0.000	1.027
Factor14	−0.012	0.014	−0.001	1.026
Factor15	−0.027	0.016	−0.002	1.025
Factor16	−0.042	0.021	−0.003	1.022
Factor17	−0.063	0.011	−0.004	1.018
Factor18	−0.074	0.025	−0.004	1.014
Factor19	−0.099	0.035	−0.006	1.008
Factor20	−0.134	.	−0.008	1.000

Factor Analysis/Correlation, Method: Principal Factors, Rotation: (Unrotated).

**Table 3:** Factor loadings (pattern matrix) and unique variances.

**Variable**	**Factor1**	**Factor2**	**Factor3**	**Uniqueness**
RGGR	0.781	−0.332	0.063	0.097
ADR	−0.343	0.776	0.023	−0.002
DGCHE	0.475	−0.411	−0.161	0.277
HCI	0.768	0.346	−0.182	0.092
NM	0.604	0.151	−0.032	0.346
GE	0.881	0.290	−0.001	0.062
CCOR	0.786	0.432	0.072	0.061
PS	0.409	−0.631	0.178	0.163
RQ	0.670	0.352	0.266	0.106
RL	0.884	0.315	0.076	0.044
PUSS	0.276	−0.166	−0.506	0.206
PH	0.262	−0.078	0.503	0.242
ABH	−0.302	−0.106	−0.504	0.141
AD	−0.343	0.776	0.023	−0.002
PDAP	−0.112	−0.249	−0.746	0.114
DR	−0.381	−0.210	0.765	0.054
GINI	−0.678	0.058	0.442	0.147
UL	−0.342	0.304	−0.182	0.416
POP	0.202	−0.422	0.731	0.074
LFIE	−0.088	0.772	0.213	0.166
**Factor**	**Variance**	**Difference**	**Proportion**	**Cumulative**
F1	5.75586	2.10800	0.5140	0.2840
F2	1.64786	0.50321	0.2081	0.4422
F3	1.14465	…	0.1281	0.5702

## Data Availability

Data of the retrieved studies are shown in Appendix A.

## Appendix A. Variables Employed in the Analysis

**Table A1:** The results of mixed-effect model.

Name		Code	Source	Definition
**Variables employed in the regression analysis**				
Life expectancy		LE	Eurostat Database [67]	Life expectancy at birth indicates the overall mortality level of a population. It summarizes the average number of years that a new born could expect to live if prevailing patterns of mortality at the time of its birth were to stay the same throughout its life
Infant mortality		MRI	Eurostat Database [67]	The number of children dying before reaching one year of age, expressed as a rate per 1000 live births in a given year
Public health expenditure (% of Total health expenditure)		CHE	Eurostat Database [67]	The overall public health spending as a percentage of total government health expenditure, referring to central and local authorities, health boards and social insurance institutions
Real GDP per capita		GDPCAP	Eurostat Database [67]	The total economic output of the country divided by the number of people and adjusted for inflation
Gini coefficient		*GINI*	World Bank Database [68]	Measure the degree of inequality in countries health and the deviation of income distribution from totally equal distribution
Unemployment rate		UL	Eurostat Database [67]	Unemployment, total (% of total labor force)
Te quality of life and dimension of governance		*F*1	World Bank Database [68]	Using data provided by World Bank, the indicator was computed based on the factor analysis method and explain x% of the total variation
Health care system performance		*F*2	World Bank Database [68]	Using data provided by World Bank, the indicator was computed based on the factor analysis method and explain x% of the total variation,
Socioeconomic vulnerabilities		*F*3	World Bank Database [68]	Using data provided by World Bank, the indicator was computed based on the factor analysis method and explain x% of the total variation
**Variables employed in the factor analysis**				
The quality of life and dimension of governance (F1)	Real GDP growth rate	RGGR	World Bank Database [68]	Real GDP growth rate (percentage change from the previous year)
	Age dependency ratio	ADR	World Bank Database [68]	Age dependency ratio, young (% of working-age population), indicates the proportion of dependents per 100 working-age population
	Domestic general government health expenditure	DGGE	World Bank Database [68]	Public expenditure on health from domestic sources % of total general government expenditure, reveal the priority of the government to spend on health from own domestic public resources
	Human Capital Index	HCI	World Bank Database [68]	Measures the amount of human capital that a child born today can expect to attain by age 18, given the risks of poor health and poor education that prevail in the country where she lives
	Nurses and midwives	NM	World Bank Database [68]	Nurses and midwives (per 1000 people) needed to provide adequate coverage with primary care interventions
	Government Effectiveness	GE	World Bank Database [68]	Measure the government implication on political social and administrative level, by capture the perceptions of the quality of public services, the quality of policy formulation and implementation, the degree of its independence from political pressures.
	Control of Corruption	CCOR	World Bank Database [68]	Capture the implication of bureaucratic regulation exercised for private gain
	Political Stability and Absence of Violence/Terrorism	PS	World Bank Database [68]	Measures perceptions of the likelihood of political instability and/or politically-motivated violence, including terrorism.
	Regulatory Quality	RQ	World Bank Database [68]	Measure the government performance in implement sound policies and regulations, capable to enhance private sector development
	Rule of Law	RL	World Bank Database [68]	Measure the agents confidence in respect the rules of society, including the the quality of contract enforcement, property rights, the likelihood of crime and violence.
	People using safely managed sanitation services	PUSS	World Bank Database [68]	People using safely managed sanitation services (% of population)
Health care system performance (F2)	Physicians	PH	World Bank Database [68]	Indicate the number of practicing physicians per 1000 population
	Available beds in hospitals	ABH	World Bank Database [68]	Avaible hospital beds which are available for the care of admitted patients
	Age dependency ratio	AD	World Bank Database [68]	Age dependency ratio, young (% of working-age population), indicates the proportion of dependents per 100 working-age population
	Premature deaths	PDAP	World Bank Database [68]	Premature deaths, % total premature deaths Ambient Particulate Matter
	Death rate	DR	World Bank Database [68]	Death rate, crude (per 1000 people)
Socioeconomic vulnerabilities (F3)	*GINI* INDEX	*GINI*	World Bank Database [68]	Standardized gini variable which measures the income inequality. The indicator was retrieved from World Bank database [68] and Smeeding and Latner [69]
	Unemployment	UL	World Bank Database [68]	Unemployment, total (% of total labor force)
	Population	POP	World Bank Database [68]	Population ages 65 and above (% of total population)
	Labor force with intermediate education	LFIE	World Bank Database [68]	Labor force with intermediate education (% of total working-age population with intermediate education)
